# Mechanical characterization of carbon nanomembranes from self-assembled monolayers

**DOI:** 10.3762/bjnano.2.92

**Published:** 2011-12-20

**Authors:** Xianghui Zhang, André Beyer, Armin Gölzhäuser

**Affiliations:** 1 Department of Physics, Physics of Supramolecular Systems and Surfaces, Bielefeld University, 33615 Bielefeld, Germany

**Keywords:** bulge test, carbon nanomembrane, mechanical characterization, self-assembled monolayers, two-dimensional materials

## Abstract

This paper reports on the mechanical characterization of carbon nanomembranes (CNMs) with a thickness of 1 nm that are fabricated by electron-induced crosslinking of aromatic self-assembled monolayers (SAMs). A novel type of in situ bulge test employing an atomic force microscope (AFM) is utilized to investigate their mechanical properties. A series of biphenyl-based molecules with different types of terminal and/or anchor groups were used to prepare the CNMs, such as 4'-[(3-trimethoxysilyl)propoxy]-[1,1'-biphenyl]-4-carbonitrile (CBPS), 1,1'-biphenyl-4-thiol (BPT) and 4-nitro-1,1'-biphenyl-4-thiol (NBPT). The elastic properties, viscoelastic behaviors and ultimate tensile strength of these biphenyl-based CNMs are investigated and discussed.

## Introduction

Ultrathin freestanding nanomembranes have recently attracted much attention as promising materials in nanotechnology [1–2]. They can be made with molecular or atomic thickness and macroscopic size, constituting two-dimensional (2-D) objects of fundamental interest as well as being suitable for applications. To this end, the mechanical stability is crucial for the fabrication of miniature yet highly sensitive nanodevices from freestanding nanomembranes. A variety of approaches to fabricate nanomembranes has been tested: Spin-assisted layer-by-layer (LBL) assembly [3–4]; spin-coating of organic–inorganic hybrid films with an interpenetrating network (IPN) structure [5–6]; cross-linking of ligand-stabilized nanoparticle assemblies at the fluid interfaces [7–8]. Freestanding nanomembranes with thicknesses from 20 to 70 nm were achieved by these approaches.

Eck et al. reported the fabrication of carbon nanomembranes (CNMs) with a thickness of 1 nm by electron-induced cross-linking of aromatic self-assembled monolayers (SAMs) [9]. Freestanding CNMs were fabricated after the dissolution of the substrate on which the SAMs were formed. A subsequent transfer with the aid of a polymeric transfer medium allowed the placement of CNMs onto arbitrary materials [10–11]. CNMs have been utilized as supporting material in transmission electron microscopy (TEM), which thus allows higher-contrast imaging of nanosized objects [11].

The mechanical properties of ultrathin nanomembranes are of particular interest as they will determine their applicability as filters, sensors or actuators. For some of the above-mentioned nanomembranes, elastic properties and tensile strength have been investigated by bulge tests [3–5]. Bulge testing is widely used to characterize the mechanical properties of freestanding films. The technique involves the clamping of a freestanding membrane over an orifice and the application of an overpressure to one side. The Young’s modulus and the prestress are then calculated from the obtained pressure–deflection relationship. The deflection is usually monitored with an optical microscope, either by viewing the membrane from the side [12] or by using a laser interferometer [13]. Both methods have a resolution in the range of hundreds of nanometers. Atomic force microscopy (AFM) has also been used for indentation studies on soft [14] as well as stiff [15] membranes. In addition, it was recently reported that the curvature of a bulged membrane was determined by AFM, while its deflection was measured with a laser sensor [16]. An optical detection of CNMs is not feasible due to their thickness of only 1 nm. However, it is straightforward to perform a complete bulge test with an AFM deflection measurement and thus to improve the resolution such that bulge testing becomes practicable for the investigation of ultrathin CNMs [10].

Here we report the mechanical characterization of one-nanometer-thick freestanding CNMs by means of bulge testing in an AFM. The AFM is used to measure the deflection of the membrane center, either by scanning a bulged membrane (the line-scanning method), or by approaching the center of the membrane and measuring the corresponding deflection (the central-point method). These techniques can be used to determine Young’s modulus and the prestress. They also allow us to investigate the viscoelastic behavior and thus generate insights into the mechanics of CNMs.

## Results and Discussion

**Figure 1:**
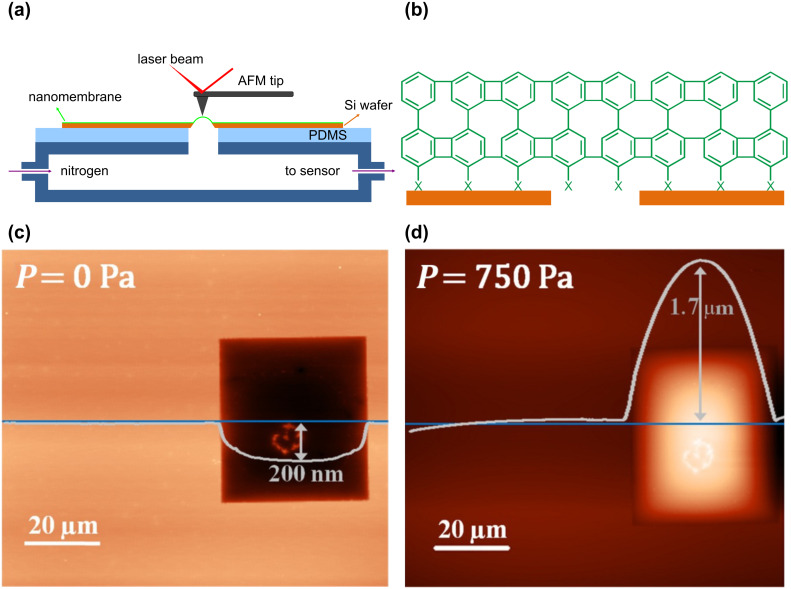
(a) Schematic diagram of a bulge test in AFM; (b) Schematic of a biphenylthiol CNM on a window-structured Si substrate, which is suspended over an orifice; (c) AFM image of a nonpressurized CNM in contact mode and the line profile with a downward deformation of 200 nm; (d) AFM image of the same membrane with an applied pressure of 750 Pa and a line profile with an upward deflection of 1.7 μm.

Figure 1a shows a schematic diagram of bulge test in an atomic force microscope. Loading of the membrane is achieved by applying a nitrogen gas pressure to the membrane. The pressure difference between the top and the bottom of the membrane is read by a pressure sensor, and the resulting deflection at the center of the membrane is recorded by an AFM tip. Figure 1b shows the scheme of a CNM that is suspended over an orifice. The high mechanical stability of CNMs allows both tapping and contact-mode scanning. Figure 1c shows a topographic contact-mode AFM image of a nonpressurized CNM, which was prepared on a rectangular opening in a silicon substrate by using the procedure described previously [9]. The CNM was formed from a self-assembled monolayer of 4'-[(3-trimethoxysilyl)propoxy]-[1,1'-biphenyl]-4-carbonitrile (CBPS) on silicon nitride membranes, which was cross-linked with a dose of 60 mC·cm^−2^ electrons. A downward step height of ~200 nm was observed due to the point load of the tip. This step height increases with the force applied by the tip. Figure 1d shows the same membrane with an applied pressure of ~750 Pa. An upward deflection of 1.7 µm was measured at the center of the membrane. Comparable images were retrieved from biphenylthiol CNMs, which were prepared by transferring the cross-linked SAMs onto window-structured silicon samples [10]. Note that the interfacial adhesion between the CNM and the substrate is mainly due to van-der-Waals contributions. Especially in the case of biphenylthiol CNMs, chemical bonds between the CNM and silicon are unlikely, as intermolecular disulfide bonds form immediately after the cleavage of the thiol-CNM from its original gold substrate. Because flexible CNMs may even conform to surfaces with a nanoscale roughness, the adhesion energy is enhanced due to an increased contact area. Apparently, this adhesion enhancement is sufficiently high to avoid delamination of CNMs from the silicon during gas-pressure loading, as shown by AFM images, e.g., Figure 1c,d.

The deflection of a membrane at the center is accessible from topographic AFM images such as in Figure 1c,d. However, this method of data retrieval is very time-consuming. In an earlier report [10] we restricted ourselves to recording line scans at the center of a membrane instead of recording full images for each applied pressure. A further development is presented in this work: The central-point method. In this method the AFM tip is brought into contact with the membrane at a preset force, only at the central point of the membrane. The main advantage of this method in comparison to scanning full lines is not in the saving of time but in the substantially reduced probability of membrane rupture events during data acquisition.

**Figure 2:**
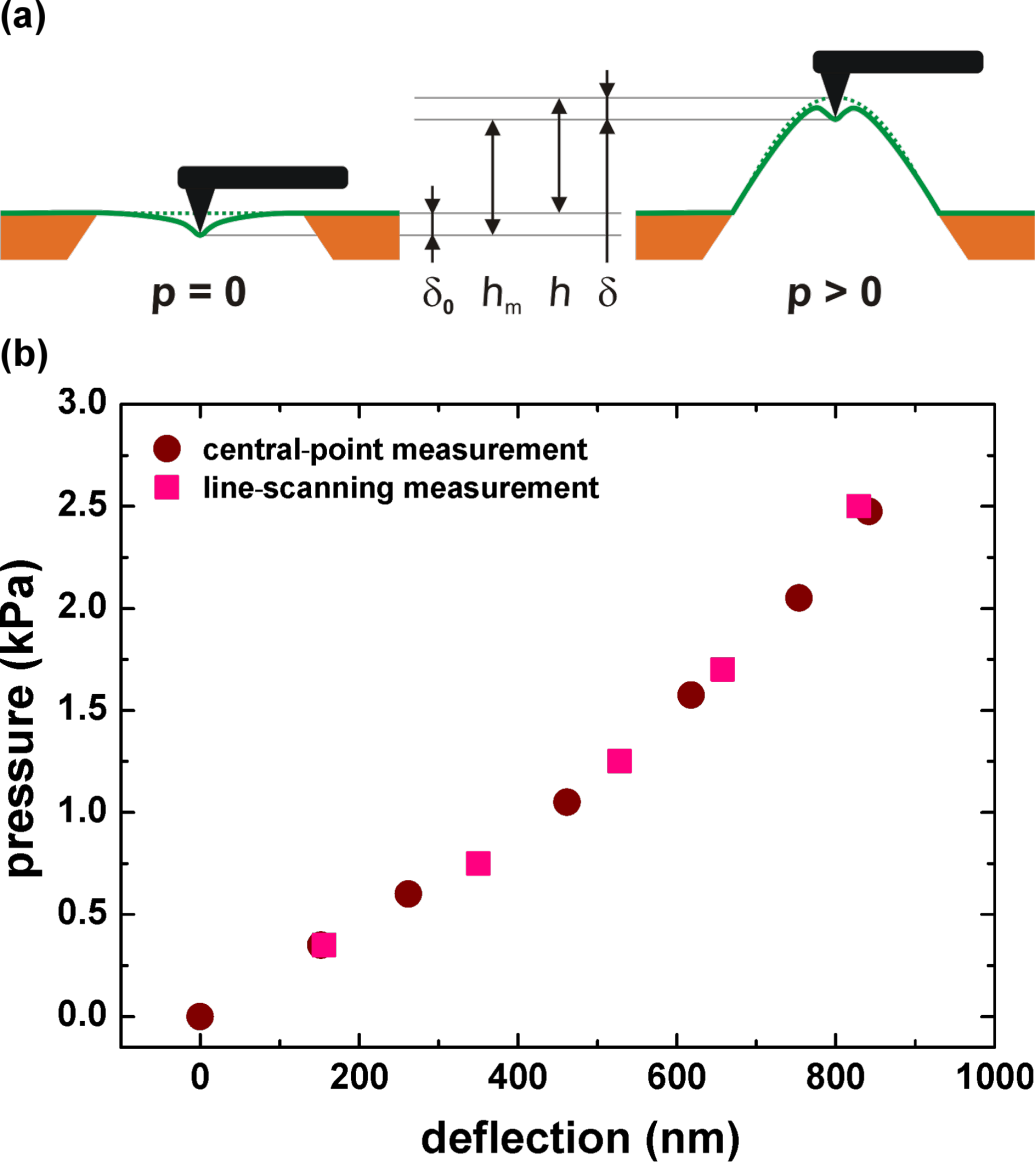
(a) Schematic of the central-point method in the bulge test; (b) Comparison of the line-scanning and the central-point method in the bulge test.

The measured deflection at the central point of a bulged membrane *h*_m_ is determined from the change of the AFM height signal due to pressurization of the membrane, as schematically shown in Figure 2a. Note that the position of the silicon frame changes when the applied pressure is varied. Therefore the AFM height signal is always measured with respect to the silicon frame. For this purpose, the AFM tip was used to probe the silicon frame near the membrane for each applied pressure. To demonstrate the feasibility of this “central-point method”, Figure 2b shows a comparison of the line-scanning method and the central-point method. It can clearly be seen that both deflection measurements are in very good agreement.

In the central-point method, the measurements are performed with a certain tip force, which is kept constant during the recording of a pressure–deflection curve. This force corresponds to an indentation depth δ_0_, which appears as a step height in topographic AFM images of nonpressurized membranes. The indentation depth δ of pressurized membranes was evaluated in order to correct the measured deflection, as described previously [10]. In this system, the tension of the CNM is assumed to be the main contribution balancing the AFM tip force. The force contributed by the bending stiffness and the adhesion between the tip and the membrane was neglected. For a pressurized membrane, the indentation depth δ decreases with increasing pressure. The change of the indentation depth Δδ is given by [10]

[1]
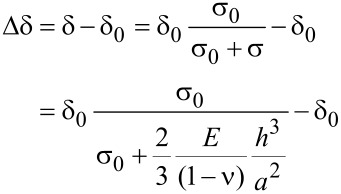


where δ_0_ is the step height in topographic AFM images of the nonpressurized membrane, *E* is the Young’s modulus, σ_0_ is the residual stress, ν is the Poisson’s ratio and 2*a* is the length of the short edge of the membrane. The corrected deflection *h* is then given by

[2]
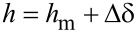


Note that Δδ is negative, i.e., the corrected deflection is always smaller than the measured value *h*_m_. This correction scheme typically results in an increase in the Young’s modulus and a decrease in the residual stress by approximately 5%.

### Elasticity

**Figure 3:**
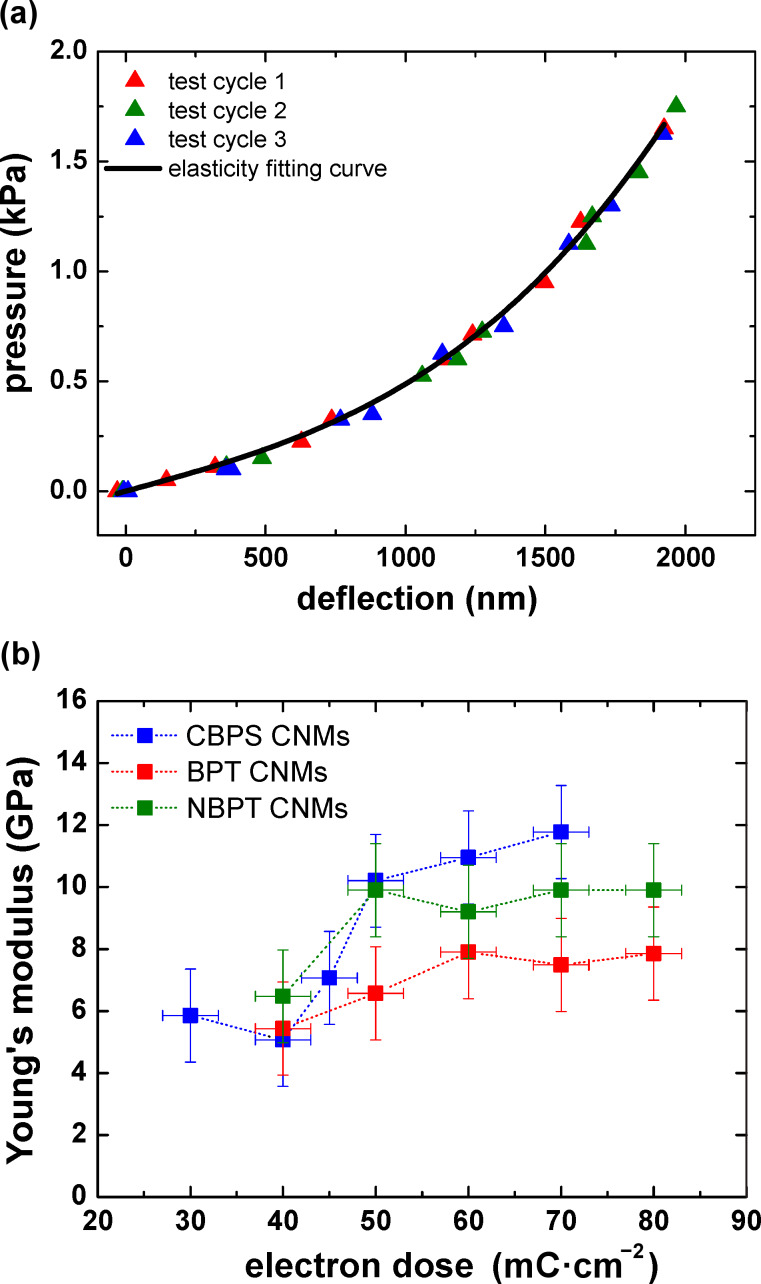
(a) Pressure–deflection relationship of an NBPT CNM with three successive loading and unloading cycles, and the corresponding elasticity fitting curve; (b) Young’s modulus for CBPS, NBPT and BPT CNMs as a function of electron irradiation doses.

In a bulge test, the elastic response is derived from the relationship between the loading pressure *p* and the resulting deflection at the center of the membrane *h*. Three successive loading and unloading test cycles were applied to a CNM with a maximum strain of ~0.66%, as shown in Figure 3a. For such deformations the membrane displays elastic behavior with a very small hysteresis of less than 5%. The relationship between pressure and deflection was derived by Vlassak and Nix [17], and an analytical formula for square and rectangular membranes is given by

[3]
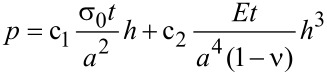


where the applied pressure *p* is a function of the corrected deflection at the center of the membrane *h*. The membrane sizes were measured in a scanning electron microscope (SEM). The constants c_1_ and c_2_ were taken from the literature [17]. The Young’s modulus *E* and the residual stress σ_0_ are accessible by fitting the above equation to the measured data.

Three different biphenyl molecules were used to fabricate CNMs. SAMs of 4'-[(3-trimethoxysilyl)propoxy]-[1,1'-biphenyl]-4-carbonitrile (CBPS) were formed on silicon nitride, SAMs of 4'-nitro-1,1'-biphenyl-4-thiol (NBPT) and 1,1'-biphenyl-4-thiol (BPT) on gold surfaces. The thickness of the respective SAMs was determined by X-ray photoelectron spectroscopy (XPS) to be ~1.6 nm for CBPS SAMs, which was larger than that of NBPT SAMs (~1.2 nm) and BPT SAMs (~0.9 nm) [9,18–19]. As cross-linking occurs between the phenyl rings, a comparable thickness is expected for the corresponding CNMs. The CNM can be modeled as a composite layer with ~1 nm thick part containing cross-linked biphenyl rings and other parts containing merely hydrocarbon chains with no contribution to the elasticity. Therefore the mechanical properties of all CNMs were evaluated by taking the same thickness of 1 nm. Figure 3b shows the evolution of the CNM elasticity during the cross-linking process, i.e., a plot of Young’s modulus of CNMs as a function of electron doses. Below 20 mC·cm^−2^, only a few intact membranes are built, indicating that the number of cross-links in aromatic SAMs is too small to allow a reliable formation of freely suspended CNMs. For electron doses between 30 mC·cm^−2^ and 50 mC·cm^−2^, more cross-links are formed and the mechanical stiffness is consequently enhanced, which facilitates the formation of freestanding CNMs. With further exposure, the Young’s moduli remained constant, even when the membrane was exposed to much higher doses, up to 80 mC·cm^−2^ (cf. Figure 3b). This behavior is in accordance with an earlier study on the thermal stability of CNMs, which indicated almost complete cross-linking at an electron dose of ~45 mC·cm^−2^ [20]. Fully cross-linked BPT and NBPT CNMs that were made on a gold substrate had a Young’s modulus of 6–8 GPa and 8–10 GPa, respectively. CBPS CNMs that were formed from a SAM on silicon nitride showed a similar elastic behavior with a Young’s modulus of 10–12 GPa. Note that CBPS SAMs are distinct from BPT and NBPT SAMs not only in the substrate, but also in the head group. Furthermore, CBPS CNMs were fabricated by direct dissolution of a 30 nm thick silicon nitride membrane without a transfer process. Conversely, for the fabrication of BPT and NBPT CNMs, it is necessary to transfer the CNM from a flat gold surface onto a silicon window. It was reported earlier that the degradation of alkanethiolate SAMs due to electrons is strongly dependent on the electrical conductivity of the substrate [21]; however, we observed the same dose dependence for both types of biphenyl-based CNMs, indicating that the conductivity of the substrate is less important. From the above, we can conclude that the elastic properties of the CNMs are mainly determined by the cross-linked aromatic units, and are independent of the type of substrate, head group or the transfer process.

Residual stresses of the CNMs were tensile in nature and varied from 40 to 120 MPa, and the residual strains varied from 0.4 to 1.6%. There was no obvious dependence on the electron dose, cf. Table 1. For CNMs, the stress is likely to be introduced during the cross-linking, as new covalent bonds are created. Obviously, the strain release is precluded due to the adhesion of the CNMs to the substrate or the polymeric transfer medium.

**Table 1:** Residual stress and strain of CNMs with different electron doses.

electron dose	30 mC·cm^−2^	40 mC·cm^−2^	50 mC·cm^−2^	60 mC·cm^−2^	70 mC·cm^−2^	80 mC·cm^−2^

BPT CNMs	–	75 MPa (1.6%)	75 MPa (1.2%)	95 MPa (1.2%)	46 MPa (0.64%)	51 MPa (0.67%)
NBPT CNMs	–	59 MPa (0.91%)	87 MPa (0.87%)	124 MPa (1.35%)	72 MPa (0.73%)	108 MPa (1.1%)
CBPS CNMs	71 MPa (1.2%)	57 MPa (1.1%)	53 MPa (0.5%)	44 MPa (0.4%)	57 MPa (0.5%)	–

### Viscoelasticity

**Figure 4:**
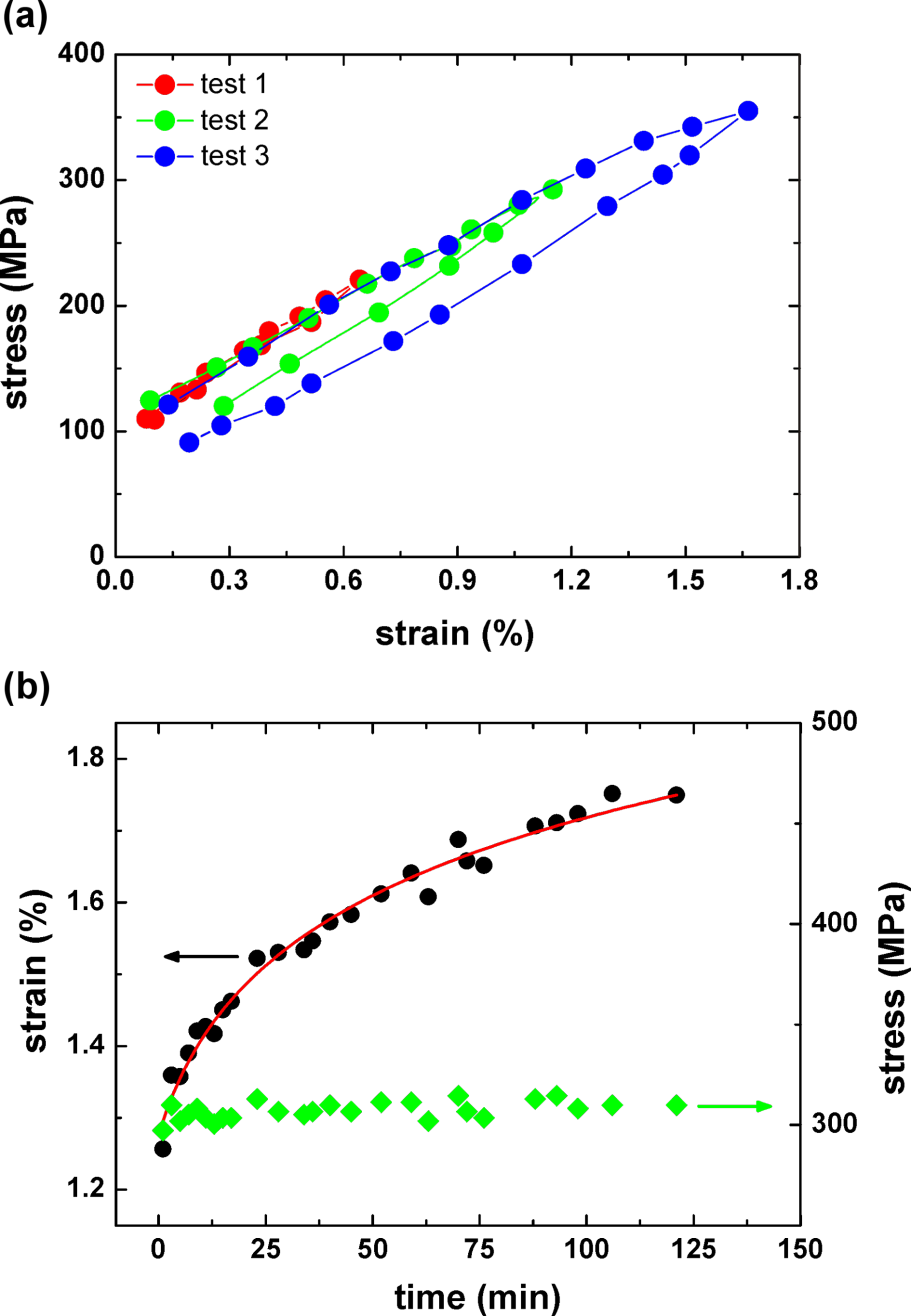
(a) Stress–strain relationship of three loading–unloading measurements on a NBPT CNM with different maximum strains at ~0.65%, ~1.2% and ~1.7%; (b) Strain exhibits a nonlinear increase at a stress of 304 ± 15 MPa, indicating a tensile-creep behavior.

Macroscopic viscoelasticity and local viscoelastic properties of soft materials have been intensively studied, for example in polymer networks or in nuclei of biological cells [22–23]. Gaining new insights into the viscoelastic behavior of one-nanometer-thick membranes requires a method with sufficient sensitivity as to determine the time-dependent deformation under a constant load. With the bulge-test setup we can perform quantitative measurements at room temperature. Figure 4a shows stress–strain curves from loading–unloading measurement cycles, with successively increasing maximum strain values of ~0.65%, ~1.2% and ~1.7%. The hysteresis loop becomes more and more pronounced with the increase in the maximum tensile strain of each cycle. Hysteresis is one major characteristic of viscoelasticity and is associated with the energy that is dissipated as heat in the loading cycles. The specific damping capacity is thus calculated based on the ratio of energy dissipated to energy stored, and the corresponding values are ~3.1%, ~9.8% and ~17.6%, respectively.

**Figure 5:**
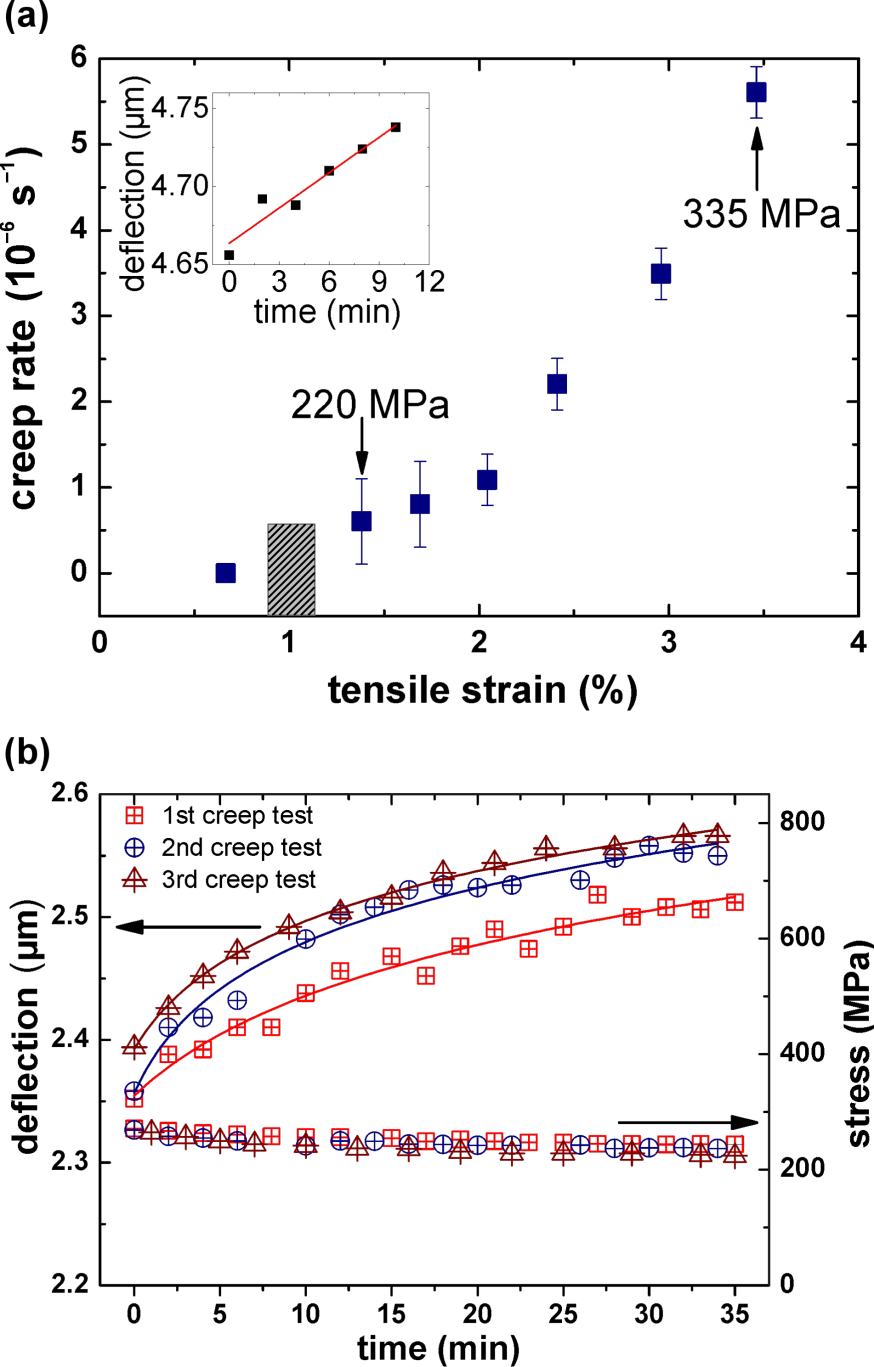
(a) Creep rate as a function of tensile strain; creep deformation can be only observed above a certain strain, e.g., ~1%. Inset: The deformation at the beginning of creep has a linear characteristic. (b) Three creep deformations were recorded at room temperature, the second test was performed 200 min after the first unloading, and the third test was performed 160 min after the second unloading.

When a CNM was loaded at a lower stress (~163 MPa), the deflection remained constant over time. However, when it was loaded at a higher stress (304 ± 15 MPa) the deformation exhibited a nonlinear increase, and thus this indicates tensile creep, as shown in Figure 4b. Note that delamination of CNMs would lead to a steplike increased deflection, but here we observed a continuous increase, indicating a strong adhesion between the CNM and the silicon. The applied strain at which creep deformation starts for CNMs is in the range of 0.8–1.2%. Strain rates as low as 10^−8^ s^−1^ can be measured with the employed AFM setup. At the beginning of loading, a linear relationship between strain and time was observed, as shown in the inset of Figure 5a. Initial creep rates were thus derived from the slopes of linear curve fits, and they increased with increasing tensile strain. As plotted in Figure 5a, initial creep rates are in the range of 10^−6^ s^−1^. This is in contrast to some polymers whose creep rate can span several orders of magnitude under different stress levels [24]. CNMs possess rather stable initial creep rates, indicating higher resistance against the creep deformation.

In order to understand its reversibility, we also employed several creep tests on a CNM and examined its recovery from previous creep deformations. Three creep tests with the same initial stress of 260 MPa were presented in Figure 5b, with the second and third creep tests carried out 200 and 160 min after the previous test, respectively. The measurements show an almost complete recovery after each test cycle. The creep behavior is a manifestation of molecular rearrangements in CNMs around defects and molecular domains, caused by stress-dependent thermal activation, and which partially recover in the absence of an external load.

### Ultimate tensile strength

**Figure 6:**
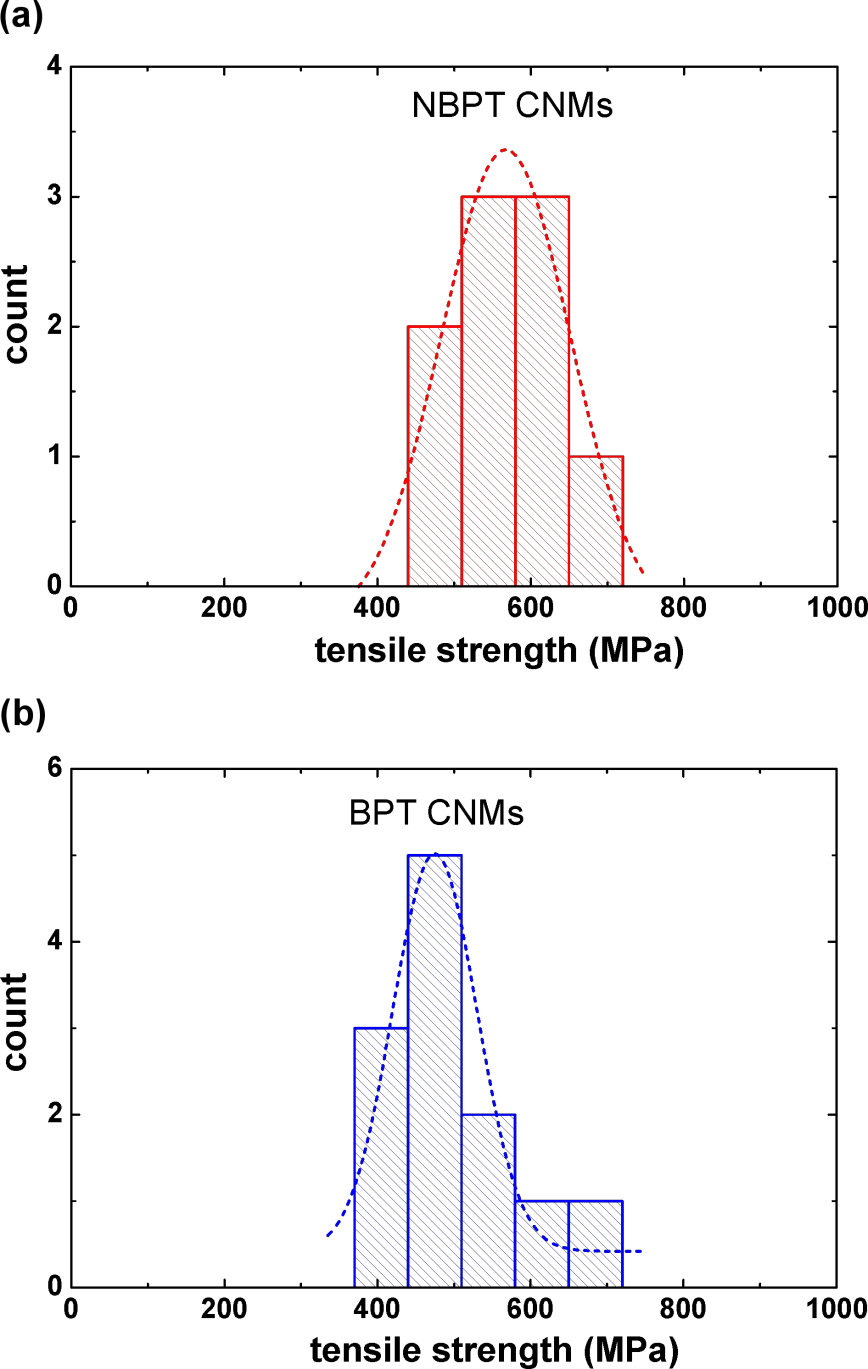
(a) Histogram of ultimate tensile strength of circular NBPT CNMs, with a peak at ~567 MPa (Gaussian peak fitting); (b) Histogram of ultimate tensile strength of circular BPT CNMs, with a peak at ~475 MPa.

Finally, we determined the ultimate tensile strength of CNMs by means of bulge tests. Rupture occurs usually at very high pressures and the corresponding deflection cannot be directly measured. The deflection is thus calculated from Equation 3. The ultimate tensile stress σ_u_ of rectangular membranes is presented as follows [13,17]:

[4]
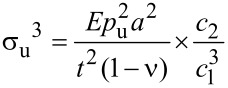


where *p*_u_ is the ultimate pressure at which the membrane ruptures. All other quantities are the same as in Equation 3. To minimize the deviation caused by different geometries of CNMs, we only selected circular membranes for the rupture tests. Equation 4 is valid for circular membranes as well [25] but with a constant value for the ratio c_2_/c_1_^3^ = 1/24. Figure 6 shows the statistical histogram of tensile strength of nine NBPT CNMs and 12 BPT CNMs. The tensile strength of NBPT CNMs ranges from 440–720 MPa with a peak located at ~567 MPa. The tensile strength of BPT CNMs has a wider distribution, with a dominating peak at ~475 MPa. These results show that NBPT CNMs possess a higher mechanical stability than BPT CNMs do, which may be caused by a higher molecular packing density in NBPT CNMs. Compared to other nanomembranes, such as IPN nanocomposite with organic–inorganic networks, which exhibit a tensile strength of 105 MPa [5], the ultimate tensile strength of CNMs is 5–6 times higher.

## Conclusion

Freestanding CNMs with 1 nm thickness were prepared from cross-linked biphenyl-based self-assembled monolayers. We employed bulge testing in order to obtain the mechanical properties of these CNMs. The preparation of fully cross-linked CNMs requires an electron dose of at least 50 mC·cm^−2^. Viscoelastic behavior in CNMs was investigated quantitatively and the results show that CNMs exhibit a high resistance against creep deformation. It was demonstrated that CNMs display a remarkable ultimate tensile strength. The molecular thickness as well as the outstanding performance in the mechanical stability enables CNMs to work in a variety of applications, e.g., as ultrathin support films in electron microscopy, as filter membranes or as highly sensitive and mechanically stable miniature transducers.

## Experimental

To prepare 4'-[(3-trimethoxysilyl)propoxy]-[1,1'-biphenyl]-4-carbonitrile (CBPS) SAMs, we used 30 nm thick silicon nitride membranes on window-structured silicon substrates (Silson Ltd., UK). The membranes were cleaned with Piranha solution (H_2_SO_4_/H_2_O_2_ in volume ratio of 3:1) for 20 min to remove organic residues. Afterwards the membranes were immersed into a ~10 mL solution of dry and degassed toluene with 10 mmol CBPS molecules for 120 h in a sealed flask under nitrogen atmosphere. For the preparation of 1,1'-biphenyl-4-thiol (BPT) self-assembled monolayers (SAMs) and 4'-nitro-1,1'-biphenyl-4-thiol (NBPT) SAMs, we use a 300 nm polycrystalline Au layer with (111) crystal planes epitaxially grown on a mica substrate (Georg Albert Physical Vapor Deposition). The substrate was cleaned with a UV/ozone cleaner (UVOH 150 LAB FHR), rinsed with ethanol and then blown dry under a nitrogen stream. Afterwards the substrates were immersed into a ~10 mmol solution of dry and degassed dimethylformamide (DMF) with 10 mmol BPT or NBPT molecules for 72 h in a sealed flask under nitrogen atmosphere.

Cross-linking was achieved in high vacuum (<5 × 10^−8^ mbar) with an electron flood gun at an electron energy of 100 eV and a current of 3 mA. Freestanding CBPS CNMs were obtained by dissolving the Si_3_N_4_ membranes on a window-structured silicon substrate (Silson Ltd., UK) in hydrofluoric acid (HF, ~48%). For BPT and NBPT CNMs, the samples were spin-coated with a layer of poly(methyl methacrylate) (PMMA) for stabilization and baked on a hotplate. The sample was immersed into HF (~48%) for 20 min to weaken the adhesion between the gold and the mica. The separation of the PMMA/CNM/Au layer from the mica was achieved by careful dipping of the sample into water. Subsequently, the Au layer was completely etched by a gold etchant (5 wt % I_2_ and 10 wt % KI in water). Afterwards, the CNM/PMMA layer was transferred to a silicon substrate with window-structured openings (Silson Ltd., UK), which was followed by dissolution of the PMMA in acetone and drying with a critical-point dryer (Autosamdri-815B, Tousimis, USA) to yield clean and suspended CNMs.

The mechanical characterization was carried out by means of bulge testing in an AFM (NTEGRA, NT-MDT, Russia). The pressure cell was made from a hollow steel cylinder with two side openings for applying and measuring the gas pressure, and one circular opening at the topside, which was sealed by the membrane. In order to establish a gas-tight connection between the membrane and the pressure cell, a layer of polydimethylsiloxane (PDMS) with a thickness of 2 mm was prepared on top of the pressure cell. The deflection at the center of the membrane was recorded by scanning the membrane with AFM in the contact mode.

In the central-point method, the AFM tip was positioned on the membrane’s center to detect the deflection of the membrane. The center was determined by measuring the position of the four edges by AFM. To this end, the tip was approached several times near an edge. The difference in the AFM height signal upon contacting the silicon frame or the freestanding CNM is easily distinguished. For each applied pressure, the AFM height signal at the center of the membrane as well as at three points on the silicon frame was measured. The measurements on the silicon were taken in order to correct any movement of the silicon frame, i.e., any change in the height position or tilt.
